# An analysis of the IS*6*/IS*26* family of insertion sequences: is it a single family?

**DOI:** 10.1099/mgen.0.000291

**Published:** 2019-09-05

**Authors:** Christopher J. Harmer, Ruth M. Hall

**Affiliations:** ^1^​ School of Life and Environmental Sciences, University of Sydney, NSW 2006, Australia

**Keywords:** insertion sequence, IS*26*, ISFinder, phylogenetic analysis

## Abstract

The relationships within a curated set of 112 insertion sequences (ISs) currently assigned to the IS*6* family, here re-named the IS*6*/IS*26* family, in the ISFinder database were examined. The encoded DDE transposases include a helix-helix-turn-helix (H-HTH) potential DNA binding domain N-terminal to the catalytic (DDE) domain, but 10 from *Clostridia* include one or two additional N-terminal domains. The transposase phylogeny clearly separated 75 derived from bacteria from 37 from archaea. The longer bacterial transposases also clustered separately. The 65 shorter bacterial transposases, including Tnp26 from IS*26*, formed six clades but share significant conservation in the H-HTH domain and in a short extension at the N-terminus, and several amino acids in the catalytic domain are completely or highly conserved. At the outer ends of these ISs, 14 bp were strongly conserved as terminal inverted repeats (TIRs) with the first two bases (GG) and the seventh base (G) present in all except one IS. The longer bacterial transposases are only distantly related to the short bacterial transposases, with only some amino acids conserved. The TIR consensus was longer and only one IS started with GG. The 37 archaeal transposases are only distantly related to either the short or the long bacterial transposases and different residues were conserved. Their TIRs are loosely related to the bacterial TIR consensus but are longer and many do not begin with GG. As they do not fit well with most bacterial ISs, the inclusion of the archaeal ISs and the longer bacterial ISs in the IS*6*/IS*26* family is not appropriate.

## Data Summary

All insertion sequences (ISs) utilized in this analysis are publicly available in the ISFinder database (https://isfinder.biotoul.fr/). The accession numbers corresponding to each of the ISs in this study are listed in Tables 3–5.

**Impact Statement**

IS*6*/IS*26* family members are known to play a key role in the dissemination of antibiotic-resistance genes. The most studied member of the family, IS*26,* performs this role in Gram-negative bacteria, and the IS*257*/IS*431* and IS*1216* groups do so in Gram-positive bacteria. This is, to the best of our knowledge, the first detailed examination of the properties of the insertion sequences (ISs) currently classified as members of the IS*6* family in ISFinder. It was undertaken to determine which ISs most resemble IS*26* and, hence, are most likely to share the unique dual mechanistic capabilities of IS*26*. In contrast to most ISs that encode a DDE transposase, IS*26* and a few related ISs have been shown to form fusions (cointegrates) between two DNA molecules rather than move as a discrete single unit. IS*26* can also catalyse a conservative reaction between two IS copies in separate DNA molecules, again forming a cointegrate. The detailed comparisons undertaken here have revealed the ISs whose transposases and ends are most closely related to those of IS*26*﻿, and identified key residues in the transposase and terminal inverted repeats that will inform future experimental studies. It has also uncovered other ISs that do not belong to this family.

## Introduction

Insertion sequences (ISs) are the simplest form of self-mobilizable mobile DNA found in bacteria and archaea. They are defined as containing only genes required for their own transposition [1, 2]. ISs were first discovered in the late 1960s via their capacity to generate mutations [3, 4]. As sequencing became more achievable and the involvement of ISs in the movement of antibiotic-resistance determinants became more evident, in particular the importance of two ISs flanking a resistance gene in a structure widely known as a class I or compound transposon, more and more ISs were identified. Prior to 1975, only 5 ISs (IS*1,* IS*2*, IS*3*, IS*4* and IS*5*) had been named, but by 1989, over 60 ISs had been identified and many of them had been sequenced and examined (see the following reviews [5–7]). Over the next decade, as sequencing became simpler and more affordable, this number expanded rapidly to over 500, and 443 of them were grouped into 17 families [2] based on shared features, primarily the similarity of the transposase enzymes that catalyse movement [2]. The ISFinder database (https://www-is.biotoul.fr/) was established to provide a central repository for the growing number of DNA sequences for ISs [8, 9]. By 2015, the number of families had grown to 32 [8] and at present, largely due to the era of genome sequencing, the sequences of over 4000 ISs are listed in the ISFinder database, though an increasing number are not strictly ISs, e.g. miniature inverted-repeat transposable elements (MITEs) and transposons.

The majority of ISs encode a transposase that contains a DDE [often DD(35)E] motif [8]. The conserved triad of acidic amino acids [aspartic acid (D), aspartic acid (D), glutamic acid (E)] act to coordinate divalent metal cations that play a vital role in the nucleophilic attack that occurs during strand cleavage and re-joining. The DDE transposases also include a DNA binding domain, e.g. helix-turn-helix or zinc finger, and features required for multimer formation [10, 11]. ISs encoding a DDE transposase are further characterized by the sequence of the terminal inverted repeats (TIRs) found at their boundaries and the size of the target site duplication (TSD) they generate when they move to a new location. However, the relationships within only a few families have been examined in any detail and IS family groupings have evolved as more sequences have become available or more detailed analysis has been performed [12, 13].

Until recently, the IS family that includes IS*26* and the IS*257*/IS*431* and IS*1216* groups, the ISs that are most commonly found associated with antibiotic-resistance genes in resistant Gram-negative and Gram-positive bacteria, had been largely neglected. IS*6*, after which this IS family was originally named [5], was discovered in 1975 as a directly repeated region bounding a kanamycin-resistance transposon in the FII plasmid JR72 [14], though the names Tn*6* and IS*6* were not used in print until 1977 [15, 16]. However, IS*6* has never been sequenced, and IS*26,* one of several named ISs that are known to be identical or nearly identical (IS*46*, IS*140*, IS1*60*, IS*176*, IS*1936*, IS15Δ1, IS15Δ2, IS15ΔIV, etc.), and the subject of most experimental studies, has become the flagship of the family. Though the source of IS*26*, the *Proteus vulgaris* plasmid Rts1, was recovered in the late 1960s [17], the first description of IS*26* came in 1982 when the transfer of a transposon carrying the kanamycin-resistance gene (*aphA1a*) from Rts1 to phage P1 was reported [18]. Tn*2680* contained a 5 kb insert flanked by 0.8 kb direct repeats that correspond to the IS, which was named IS*26* [18]. IS*26* (GenBank accession number X00011) was sequenced in 1983 [19] and experimentally characterized in the mid-1980s [19–22]. Later, Tn*2680* was shown to be homologous to Tn*6* by restriction mapping and examination by electron microscopy of heteroduplexes [21]. Hence, while IS*6* is most likely identical or nearly identical to IS*26*, in the absence of a sequence and, hence, an ISFinder entry for IS*6*, here the family will be called the IS*6*/IS*26* family.

While many ISs transpose by either a ‘cut-out-paste-in’ or a ‘copy-out-paste-in’ mechanism [11], IS*26* uses a characteristic ‘replicative’ or ‘copy-in’ mechanism to exclusively form cointegrates rather than move alone to a new location. Apparent transposition events (a single IS*26* surrounded by an 8 bp TSD) likely arise via this route, followed by resolution of the cointegrate via homologous recombination [20]. Because of the requirement for homologous recombination, structures consisting of directly-oriented IS*26* copies flanking a passenger region cannot move as a unit and were designated ‘pseudo-compound’ transposons [5]. Some of the ISs known to be identical to or very close relatives of IS*26* (IS*46*, IS*160*, IS*15*Δ1 and IS*15*Δ2) were also shown to form cointegrates [23–27], but IS*6* was not examined. A few other more distantly related IS*6/*IS*26* family members have also been shown experimentally to form cointegrates [28–35]. The copy-in mechanism has only ever been described for members of the IS*6*/IS*26* family and the ISs related to members of the Tn*3*/Tn*21*/Tn*501*, class II transposon family, which encode a dedicated resolution enzyme and, hence, can transpose [8].

Recently, a further reaction unique to IS*26* has been documented. In order to explain the configuration of regions containing multiple IS*26* copies [36], IS*26* movement was re-examined and, in addition to the known copy-in cointegrate formation route, cointegrates can be formed using a conservative mechanism when both DNA molecules carry a copy of IS*26* [37–40]. This novel mechanism can rescue the circular products of replicative deletion events to create structures resembling transposons [38], explaining the critical role that members of this family play in shaping the evolution of Gram-negative and Gram-positive bacteria, particularly in the context of the dissemination of many different antibiotic-resistance determinants within and between bacterial species.

Given the importance of members of the IS*6*/IS*26* family in the dissemination of antibiotic-resistance genes and the renewed interest in this family, a deeper understanding of the family is needed. ISFinder is a sequence repository based on sequence data alone, making all classifications predictive. However, as the unusual copy-in (replicative) and conservative mechanisms of IS*26* are not shared with any other IS family studied to date, particular care and preferably experimental studies demonstrating cointegrate formation are needed before placing further ISs in the family.

Here, we have analysed the relationships between ISs currently classified as IS*6* family members in the ISFinder database [9]. We aimed first to identify the ISs that are sufficiently related to IS*26* to predict that they may share the same mechanistic capabilities, and second, to identify the key conserved residues in the transposase and the TIRs that may be important for the dual mechanism, and hence inform future experimental studies.

## Methods

### Databases and curation

To facilitate an accurate analysis of the entire IS*6*/IS*26* family of ISs, a stand-alone database was built by downloading sequence information on all members of the IS*6*/IS*26* family from the ISFinder (www-is.biotoul.fr) [9]) (last searched 25 February 2019). The nucleotide and protein sequences for the 151 ISFinder entries, as well two additional IS*6* family members not found in ISFinder [ISSau10 (GenBank accession no. FN390947) [41] and ISCap (GenBank accession no. EF177828) [42]], were compiled into a custom blast database using Geneious version 7.1.9 (Biomatters). The first 20 bp and the last 20 bp of each IS were also compiled into a separate custom blast database to facilitate analysis of the TIR consensuses.

A ClustalW alignment (BLOSUM62 matrix, gap open cost 10, gap extend cost 0.1) was performed to align all 153 nucleotide and protein sequences, and the pairwise identity matrix output was used to identify any pairs of ISs that violated the current ISFinder rules for defining isoforms (sharing >98 % amino acid similarity and/or >95 % nucleotide identity with any other IS in the database). These ISs (Table S1, available with the online version of this article), which are noted as isoforms in ISFinder, were removed from the database. The initial transposase alignment was inspected manually to identify any IS with a transposase that was significantly shorter than normal, that included a stop codon or that lacked one of the critical DDE residues. Alternate examples of these ISs were sought in the GenBank non-redundant nucleotide database and substituted if available. Where alternate examples for ISs with defective transposases were not available (i.e. the ISFinder entry represents the only time that IS has been sequenced), the IS was removed from the database. ISs that did not both start and end with GG were re-examined using additional available sequences, allowing the ends to be redefined by removing a single base at each end. All alterations to sequences in the custom database are listed in Tables S2 and S3. All subsequent alignments were performed using the curated data set and the same ClustalW parameters in Geneious version 7.1.9. All alignments and trees generated were exported to Adobe Illustrator (Adobe).

We elected to use IS nomenclature as follows: for ISs with only numbers, the numbers are italic, for all other IS no italics are used. Though in the tables we have used the source species found in ISFinder, some members of clade I, particularly IS*6100*, which is currently listed as derived from a *Mycobacterium fortuitum *, and IS*26* are found in several Gram-negative species via their association with rapidly evolving antibiotic-resistance regions.

### Phylogenetic analysis

A phylogenetic analysis of the transposases was performed by reconstructing a circular neighbour-joining tree using a Jukes–Cantor model on the ClustalW alignment, either unrooted or with the IS*903* transposase as an outgroup. IS*903* was chosen as an outgroup because it belongs to the separate IS*5* family but encodes a DDE transposase of similar length to those found in the IS*6* family. The analysis was also repeated using ISPfu1 as an outgroup. ISPfu1 is classified in ISFinder as an IS*6* family member and represents the most distantly-related archaeal IS, sharing the lowest identity with any of the bacterial ISs. A consensus tree was built by resampling the analysis 10 000 times.

### Secondary structure modelling

Transposase predicted secondary structures were determined by submitting the amino acid sequences to the JPred4 server (http://www.compbio.dundee.ac.uk/jpred/) using default parameters [43]. The JPred4 output was mapped onto the Geneious-generated alignments of the transposases to confirm conservation of the transposase secondary structure across clades.

## Results

### Curation of the IS*6*/IS*26* family dataset from ISFinder

In the ISFinder database, there are currently 154 entries listed under the IS*6* family (last searched 25 February 2019). However, the ISFinder entry for IS*1936* does not contain a DNA sequence. IS*15* is now known to be an IS*26* variant, IS*15*Δ1, inserted in a second IS*26* variant, IS*15*Δ2, and two MITEs, MITEBth2 and MITEHarch3, lack a transposase and cannot mobilize themselves. These three entries are not strictly ISs and were excluded from the analyses.

Not all full length entries comply with the current rules for submission, namely that only one isoform of each IS, defined as >98 % amino acid similarity for the transposase and/or >95 % nucleotide identity, can be recorded. These appear to be ISs that were submitted prior to the introduction of this isoform exclusion system, and are almost identical or closely related to another IS (Table S1). This includes IS*15*Δ1 and IS*15*Δ2, which are isoforms of IS*26*, with only 3 or 1 bp different and a single amino acid substitution in the Tnp26 transposase (see [44]). The majority of the isoforms found were isoforms of ISS1, IS*1216* and IS*257/*IS*431*, all recovered from Firmicutes (Gram-positive bacterial species) (Table S1). Exclusion of these isoforms reduced the number of ISs to 120. However, we note that isoform designation is inevitably dependent on which IS the sequence is compared to, and this is illustrated in Tables 1 and 2 for ISs related to or named as IS*257* [45] and IS*431* [46]. As both names have been used extensively, we refer to this isoform group as the IS*257/*IS*431* group. Relative to IS*257*R1, which is used by ISFinder, both the pairwise DNA identities in Table 1 and the pairwise transposase identities in Table 2 would classify all but IS*257*-2 as isoforms. However, if IS*257*-1 had been used as comparator, IS*257*-3 and IS*431*L would not be isoforms. Tables 1 and 2 also show that, despite the name, IS*257*-2 is not closely related to other members of the IS*257/*IS*431* group (86.3 % identical to IS2*57*R1). This level of divergence from members of the IS*257*/IS*431* group is similar to that of ISSau6 and we recommend that IS*257*-2 should be re-named as ISSau11.

**Table 1.** IS*257/IS431* pairwise DNA identity (%) Lighter shading denotes the >95 % nucleotide identity cut-off applied by ISFinder to define isoforms. Darker shading denotes the nucleotide identity to IS*257*R1, the IS used to define isoforms in this group. Lengths of ISs as used to calculate DNA identity: 788 bp, IS*431*L; 789 bp, IS*257*-3 and IS*257*R2; 790 bp, IS*257*-2, IS*431*R, IS*431*mec and IS*257*R1; 791 bp, IS*257*-1.

	IS*257*-2	IS*257*-1	IS*257*-3	IS*431*L	IS*257*R2	IS*257*R1	IS*431mec*	IS*431*R
IS*257*-2	100	88.86	88.83	86.15	86.29	86.31	86.82	86.57
IS*257*-1		100	92.52	94.92	94.8	95.06	95.57	95.32
IS*257*-3			100	95.3	96.7	95.18	95.69	95.44
IS*431*L				100	97.59	97.21	97.72	97.97
IS*257*R2					100	98.23	97.97	97.72
IS*257*R1						100	99.24	98.99
IS*431mec*							100	99.49
IS*431*R								100

**Table 2.** IS*257/IS431* transposase amino acid similarity (%) Lighter shading denotes the >98 % amino acid similarity cut-off applied by ISFinder to define isoforms. Darker shading denotes the amino acid similarity to IS*257*R1, the IS used to define isoforms in this group. Lengths of transposases as used to calculate amino acid similarity: 221 aa, IS*257*-2; 224 aa, IS*257*-1, IS*257*-3, IS*431*L, IS*431*R, IS*431*mec, IS*257*R1 and IS*257*R2.

	IS*257*-2	IS*257*-1	IS*257*-3	IS*431*L	IS*257*R2	IS*257*R1	IS*431mec*	IS*431*R
IS*257*-2	100	96.55	97.04	96.05	96.05	95.56	96.05	96.55
IS*257*-1		100	98.66	98.21	98.66	98.21	98.66	99.10
IS*257*-3			100	98.66	99.10	98.66	99.10	99.10
IS*431*L				100	98.66	98.21	98.66	99.55
IS*257*R2					100	99.55	99.10	98.66
IS*257*R1						100	98.55	99.10
IS*431mec*							100	99.10
IS*431*R								100

Additional ISFinder entries for bacterial ISs that did not encode a complete transposase were replaced or excluded from further analysis if alternate sequences were not available (Table S2). The ISEnfa1 entry was replaced with the sequence found in KX579977, and ISSod8, ISAcr2, ISCgl3, ISSpu17 (with truncated Tnp) and IS*240*F (internal stop codon) were removed. ISCca8 and Cca16 (missing the first D of DDE), and ISMno27, ISMno35 and ISXne2 (missing the E of DDE) were also removed. Many further ISs are represented by a single sequence in GenBank and may have either mutations or errors affecting the transposase.

Among the remaining 110 IS*6/*IS*26* family members, 73 were found in bacteria and 37 in archaea. The ISs found in Gram-negative and Gram-positive bacteria are listed in Tables 3 and 4, respectively. The ISs found in archaea are listed in Table 5. Two IS*6* family members, ISCap (GenBank accession number EF177828) and ISSau10 (GenBank accession number FN390947), that have been reported in staphylococci [41, 42] but not added to the ISFinder database, were included in the analysis, bringing the final number of ISs analysed to 112.

**Table 3.** Bacterial Gram-negative IS*6* family members analysed Only ISs conforming to the following criteria were included in this analysis: IS must follow the current ISFinder rules (IS sharing >98 % amino acid similarity and/or >95 % nucleotide identity are considered the same IS), IS must include an intact transposase, and the transposase must contain a conserved DDE catalytic triad.

IS	IS length (bp)	Tnp length (aa)	TIR (bp)*	TSD (bp)†	Clade	Accession no.‡
**Proteobacteria** **Gammaproteobacteria**						
IS*26*	820	234	14	8	I	X00011
IS*1006*	819	234	16	nd	I	NC_004361
IS*1007*	819	234	18	nd	I	AJ250860
IS*1008*	820	234	17	nd	I	AJ251307
IS*1327*	810	234	15/16	nd	IV	X87144
IS*1635*	861	245	14	nd	I	Y18002
IS*6100*§	880	254	14	8	I	X53635
ISEas1	811	231	16	nd	IV	CP011588
ISEc59	891	240	17/23	nd	VI	KX246266
ISPpr9	858	245	18/22	nd	I	NC_005871
ISPsa2	863	242	17/19	nd	V	HM563000
ISSba18	829	230	14/16	nd	IV	NC_009999
ISVsa4	861	246	15/18	nd	I	NC_011311
ISYps1	809	231	16	8	IV	FM178282||
**Proteobacteria** **Alphaproteobacteria**						
IS*2020*	815	228	16/19	nd	IV	AF118548
ISApr5	806	231	11/12	nd	III	NZ_ABHC01000011
ISBj7	824	253	14/16	nd	III	BA000040
ISMex25	887	250	13/15	nd	VI	CP001513||
ISMno28	892	250	18/21	nd	VI	NC_011892
ISMno34	889	250	14/15	nd	VI	NC_011892
ISMno36	888	250	15/18	nd	VI	NC_011892
ISMno37	889	250	25/34	nd	VI	NC_011894
ISMno6	816	233	15	nd	VI	NC_011887
ISMpo1	888	250	15/16	nd	VI	NC_010727
ISMtsp1	893	250	15	nd	VI	NZ_ABAY01000077
ISMtsp2	894	250	16/18	nd	VI	NZ_ABAT01000047
ISPko6	817	227	15/19	nd	IV	KP294352||
ISPpa9	812	236	15	nd	III	EU909903
ISPmar1	821	227	18/21	8	IV	GU997095
ISRle39A	890	250	18/22	nd	VI	X99520
ISRle6	891	256	15/17	nd	VI	NC_008382
ISRle7	891	250	14	nd	VI	NC_008382
ISRH1	809	250	15/16¶	nd	III	AF023675
ISRsp9	818	237	18/20	nd	III	FO082821||
ISRssp3	810	236	20/24	nd	III	NZ_AAMC01000007
ISStag1	889	255	17/18	nd	I	NZ_AAUW01000024
**Proteobacteria** **Betaproteobacteria**						
ISBmu21	836	240	18/24	nd	IV	AP009387
**Other phyla**#						
IS*1628*	841	236	20	nd	I	AF121000
ISAcma1	844	236	13/14¶	nd	V	NC_009932
ISCca2	834	235	12/13	8	IV	CBQZ010000005
ISCef5	843	236	21/23¶	nd	I	NC_004320
ISDge13	806	237	19	nd	III	NC_009939
ISDge15	747	230	16/17	nd	III	NC_008010
ISSus3	823	237	33/34	nd	V	NC_003106

*TIR length as recorded in ISFinder.

†TSD length as recorded in ISFinder. nd denotes no TSD observed in the sequence submitted to ISFinder, but does not preclude the presence of a TSD in other sequenced examples of that particular IS.

‡Reference sequence recorded in ISFinder.

§Origin in ISFinder is recorded as *Mycobacterium fortuitum* (Actinobacter), though this IS is overwhelmingly found in Gammaproteobacteria.

||No accession number recorded in ISFinder. Accession number identified by performing a blast search using the sequence in the ISFinder entry.

¶TIRs were incorrectly defined in ISFinder. Additional available sequences were examined, and the TIRs were modified by removing 1 G nucleotide at the left and right ends of ISCef5 and ISAcma1 (based on GenBank accession numbers CP010451 and CP000844, respectively), or by removing 1 C nucleotide at each end of ISRH1 (based on GenBank accession number NZ_PIQN00000000). The IS lengths were adjusted accordingly.

#IS*1628* and ISCef5, Actinobacteria Actinobacteria; ISCca2, Bacteroidetes Cytophagia; ISAcma1, Cyanobacteria Synococcales; ISDge13 and ISDge15, Deonococcus–Thermus Dinococci; ISSus3, Acidobacteria Solibacteres.

**Table 4.** Bacterial Gram-positive IS*6* family members analysed^*^ Only ISs conforming to the following criteria were included in this analysis: IS must follow the current ISFinder rules (IS sharing >98 % amino acid similarity and/or >95 % nucleotide identity are considered the same IS), IS must include an intact transposase, and the transposase must contain a conserved DDE catalytic triad.

IS	IS length (bp)	Tnp length (aa)	TIR (bp)*	TSD (bp)†	Clade	Accession no.‡
**Firmicutes Bacilli**						
IS*1216*E	808	226	24	nd	II	U49512
IS*240*A	861	235	16/17	nd	V	M23740
IS*240*C	817	241	16/17	nd	V	CP015730§
IS*257-2*	790	221	20/26	8	II	X53951
IS*257*R1	790	224	18/20	8	II	M13290
ISBth6	864	238	17/18	nd	V	NZ_AAJM01000806
ISBth20	808	228	16/18	nd	II	CP003766
ISBwe2	863	235	16	nd	V	NC_010180
ISBwe3	843	235	15/16	nd	V	NC_010180
ISCap	793	224	22	nd	II	EF177828
ISEnfa1||	808	228	18/19¶	nd	II	KX579977
ISLgar4	809	223	17/18	nd	II	AMFE010000010
ISLmo3	807	226	17	nd	II	CP022021
ISLmo4	812	226	17	nd	II	CP006611§
ISS1N	808	226	18	8	II	M37395
ISS1S	808	226	18	8	II	M18294
ISS1W	809	226	17/18	8	II	M37396
ISSau6	793	224	21/22	nd	II	NC_002952
ISSau10	793	225	16	8#	II	FN390947
ISTeha2	809	226	17	nd	II	AP012046
**Firmicutes Clostridia –** **c** **lade VII and solo**						
ISCbo1	1307	344	20/25	8	VII	NC_015425
ISClte3	1274	340	21/32	nd	VII	AE015927
ISCpe7	1274	340	12/15	8	VII	NC_008262
ISDsp3	1456	319	28/36	8	Solo	NC_009455
ISNth1	1226	353	17/23	8	VII	NZ_ABKR01000003
ISTps1	1349	343	18/23	8	VII	CP000924
ISTps1a	1339	343	12/16	nd	VII	NC_010320
**Firmicutes Clostridia –** **c** **lade VIII**						
ISHahy6	1713	453	14/19	8	VIII	CP002304
ISHco2	1727	453	14/19	nd	VIII	FNDF01000001
ISTet2	1761	454	15/23	8	VIII	AEYS01000011
**Actinobacteria Actinobacteria**						
ISFal2	826	236	13/19	nd	Solo	NC_008278

*TIR length as recorded in ISFinder.

†TSD length as recorded in ISFinder. nd denotes no TSD observed in the sequence submitted to ISFinder, but does not preclude the presence of a TSD in other sequenced examples of that particular IS.

‡Reference sequence recorded in ISFinder.

§No accession number recorded in ISFinder. Accession number identified by performing a blast search using the sequence in the ISFinder entry.

||The sequence in ISFinder for ISEnfa1 (GenBank accession number AY884205) included a 10 bp deletion, resulting in a truncated transposase. A second complete sequence for ISEnfa1 was identified (GenBank accession number KX579977) and used to replace the truncated sequence.

¶TIRs were incorrectly defined in ISFinder. Additional available sequences were examined, and the TIRs were modified by removing 1 G nucleotide at the left and right ends of ISEnfa1 based on GenBank accession number CP031028. The IS length was adjusted accordingly.

#No TSD recorded in ISFinder, but an 8 bp TSD was reported by Gomez-Sanz *et* *al*. in 2013.[56].

**Table 5.** Archaeal ISs analysed Only ISs conforming to the following criteria were included in this analysis: IS must follow the current ISFinder rules (IS sharing >98 % amino acid similarity and/or >95 % nucleotide identity are considered the same IS), IS must include an intact transposase, and the transposase must contain a conserved DDE catalytic triad.

IS	IS length (bp)	Tnp length (aa)	TIR (bp)*	TIR end†	TSD (bp)‡	Accession no.§
ISC735	735	214	15	TA/GA	nd	AY671942
ISC774	778	231	16	GT	nd	NC_002754
ISH14	696	211	14/15	GG	8	NC_006396
ISH15	697	211	15/17	CG/CA	9	NC_006396
ISH17	745	226	14	GG	8	NC_006393
ISH29	697	211	16/17	GG	8	NC_002608
ISHarch12	698	211	15/17	GG	8	CP002988
ISHarch13	698	211	15/17	GG	nd	CP002988
ISHarch14	698	211	15/17	GG/CA	8	CP002988
ISHarch4	743	223	18	GG	8	CP002989
ISHbo1	745	225	15	GG	8	CP001692
ISHla13	745	226	14/15	GG	nd	CP001366
ISHli8	745	226	15/16	GG	nd	CP024845||
ISHli9	742	224	18/22	GG	8	CP024845||
ISHme1	740	225	15	GG	nd	CP001871
ISHmu2	671	211	13/14	GG/GA	8	CP001688
ISHmu9	697	206	14/22	GG/GT	nd	CP001688
ISHtu2	743	225	15/18	GG	8	CP001861
ISMja1	703	214	19/22	GA	nd	NC_000909
ISNgar3	697	211	14/15	GG/CA	8	CP003377
ISNamo10	697	211	13/17	GG/TG	nd	HF582854
ISNamo11	698	211	15/17	CA/CC	8	HF582854
ISNamo12	697	211	14/17	GG	8	HF582854
ISNamo13	745	226	16/18	GG	8	HF582854
ISNamo7	697	211	14/17	GG	8	HF582854
ISNamo8	696	211	15/17	GG	nd	HF582854
ISNamo9	696	211	15/17	GG	nd	HF582854
ISNma4	744	226	12	GG	nd	CP001932
ISNpe4	696	211	12/14	GG	8	CP003372
ISNpe9	742	224	12/14	GG/GA	3	CP003372
ISNph1	697	211	15/17	GG/CA	nd	NC_007426
ISPfu1	781	233	15/16	GA	8	NC_003413
ISPfu2	782	233	15/16	GA	8	NC_003413
ISPfu5	779	233	15/16	GA	9	NC_003413
ISSis1	735	215	18	GA	8	NC_006424
ISSte1	746	217	16	GT	8	NC_005969
ISSto2	853	237	33/34	GG	5	NC_003106

*TIR length as recorded in ISFinder.

†5′ and complementary 3′ sequence.

‡TSD length as recorded in ISFinder. nd denotes no TSD observed in the sequence submitted to ISFinder, but does not preclude the presence of a TSD in other sequenced examples of that particular IS.

§Reference sequence recorded in ISFinder.

||No accession number recorded in ISFinder. Accession number identified by performing a blast search using the sequence in the ISFinder entry.

### Bacterial and archaeal transposases are distantly related

**Fig. 1.**
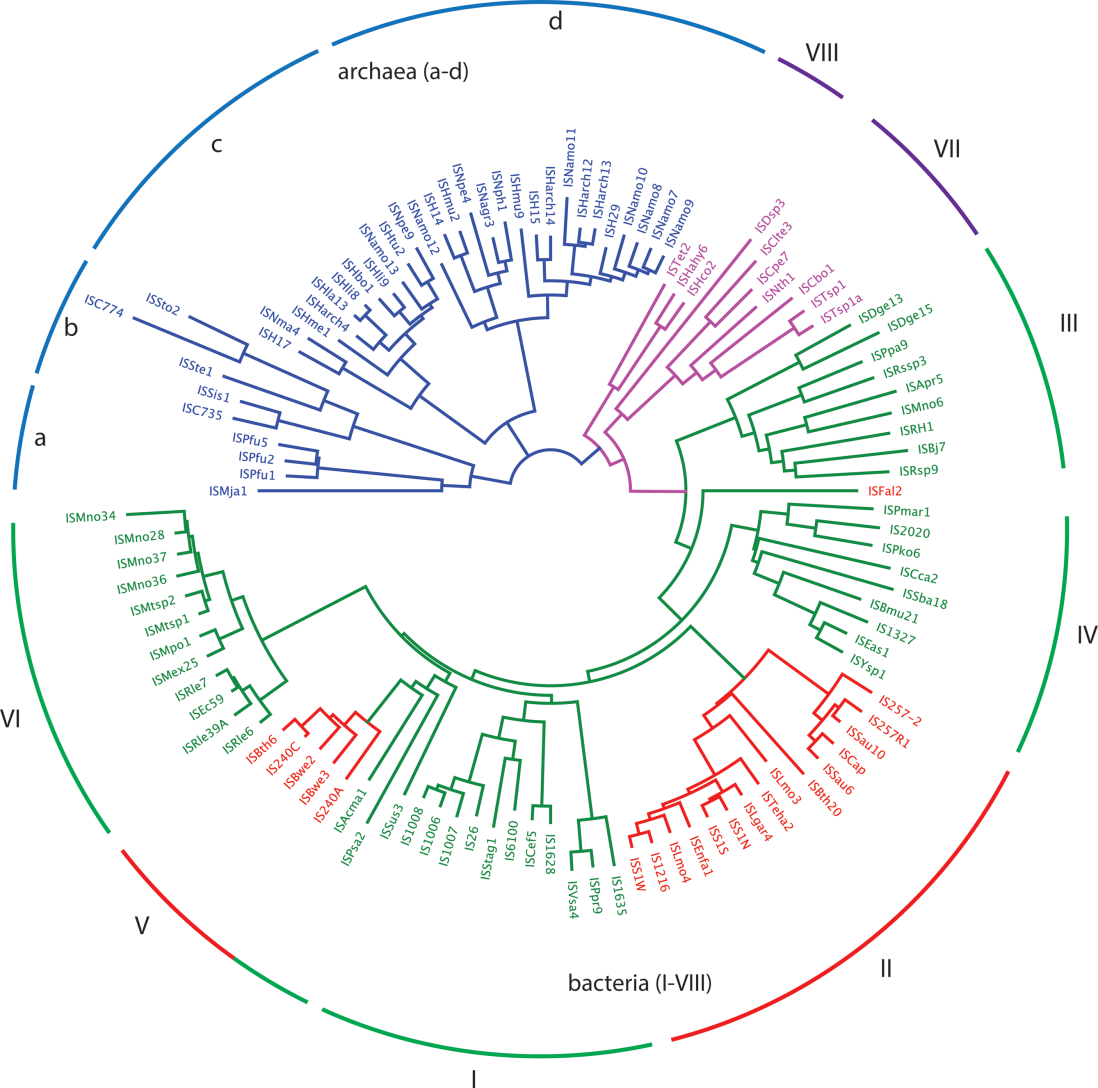
Un-rooted neighbour-joining phylogeny of 112 IS*6*/IS*26* family transposases. The consensus tree was reconstructed by resampling the analysis 10 000 times. ISs derived from bacteria are coloured green (Gram-negative), red (Gram-positive) or purple (divergent longer bacterial ISs derived from *Clostridium* species), and ISs of archaeal origin are blue. Clades encompassing ISs of bacterial origin are labelled I to VIII, and clades encompassing ISs of archaeal origin are labelled a to d.

Initially, we sought to align all members of the family in order to identify the most conserved residues and, thus, inform our ongoing experimental studies on IS*26*. However, we were unable to obtain any informative alignments and in order to understand why this was so a phylogenetic analysis of the transposases of all 112 ISs in the consolidated data set was performed by building an un-rooted neighbour-joining tree (Fig. 1). Repetition of the analysis using the transposase of either IS*903* from the IS*5* family or the archaeal putative IS*6* family member ISPfu1 as outgroup produced trees with the same topology (data not shown). The transposases fall broadly into three very distinct branches, corresponding to those from ISs found in the archaea (four clades), the majority of those from ISs found in bacteria (six clades), and a second bacterial group (two clades). Though it was recently claimed that the transposases of all bacterial members of this family listed in ISFinder share at least 40 % identity [8], when individual archaeal transposases were compared to individual bacterial transposases, pairs share no greater than 25 % amino acid identity. Hence, the ISs of archaeal origin appear to be at best distantly related to those found in bacteria and may not be family members.

**Fig. 2.**
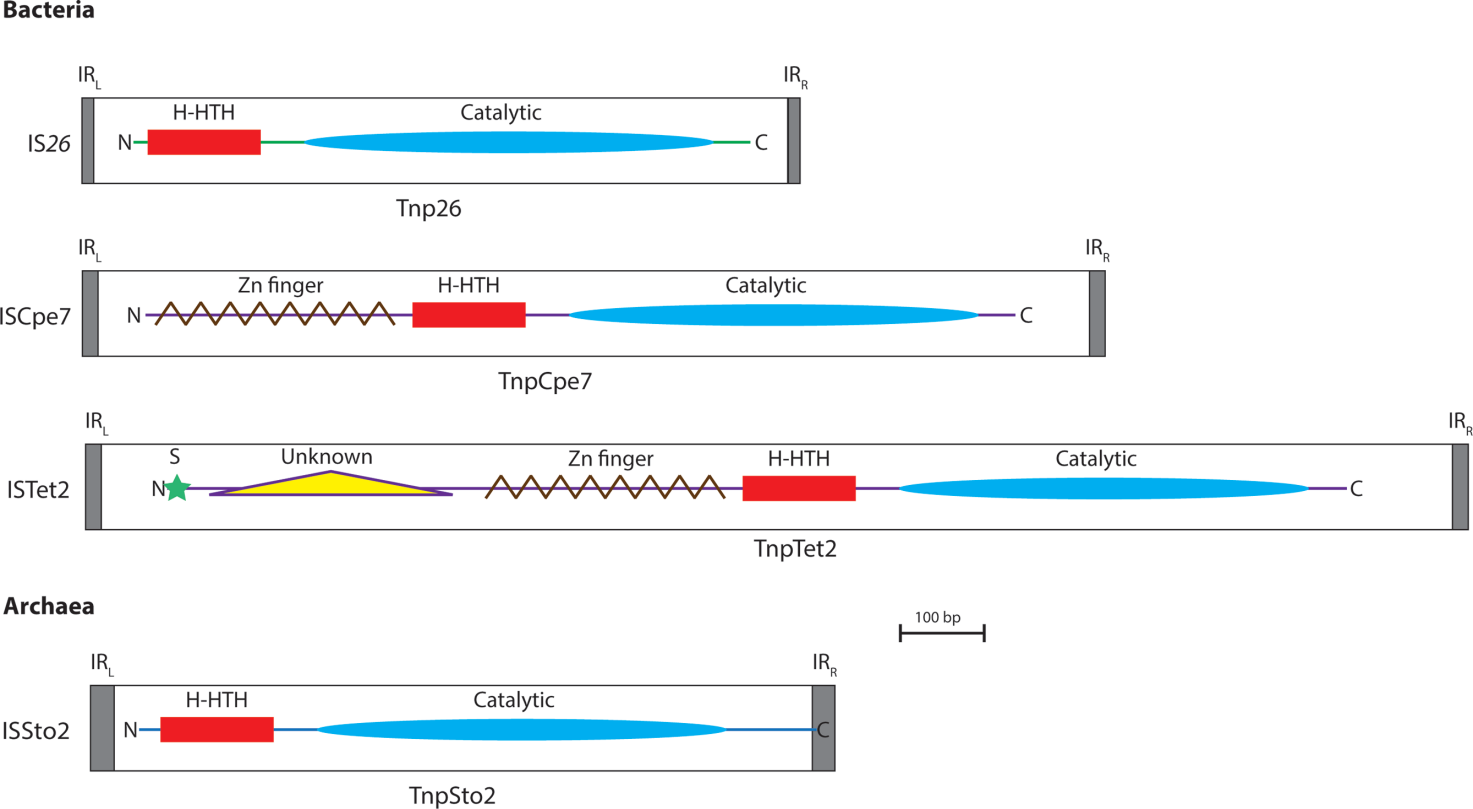
Structure of representative bacterial and archaeal IS*6*/IS*26* family members. The extent and orientation of the transposases are shown with the N-terminal (N) and C-terminal (C) marked. Protein secondary structures predicted by JPred are marked, including predicted catalytic domains (blue ovals), H-HTH motifs (red boxes), zinc fingers (brown zig-zags) and an unknown domain (yellow triangle), and a signal peptide (S, green star). TIRs are shaded in grey. Drawn to scale in Gene Construction Kit (GCK, version 4.0) from the corresponding entries in the ISFinder database before transposing into Adobe Illustrator.

The bacterial transposases in clades VII and VIII also lie on a distinct branch and are significantly larger (>1000 bp) than most of their bacterial counterparts which, as noted before [8], range in size from 789 bp (IS*257*) to 880 bp (IS*6100*). They include an N-terminal extension of approximately 90 aa that forms a zinc finger domain, also noted elsewhere [8]. Nine of the ten longer transposases were in two groups (clades VII and VIII in Fig. 1). Six ISs in clade VII (ISClte3, ISCpe7, ISCbo1, ISTsp1, ISTsp1a and ISNth1) have a larger size of 1226–1336 bp (Table 4) and are represented by ISCpe7 in Fig. 2. One, ISDsp3, at 1456 bp is of intermediate size (not shown in Fig. 2) and not in either clade, does not include an intact Zn finger domain. Three further ISs in clade VIII (ISHahy6, ISHco2 and ISTet2) are >1700 bp long, include the zinc finger domain and a further 100 aa N-terminal extension of unknown function with a signal peptide at the N-terminus (represented by ISTet2 in Fig. 2). These transposases also share very low levels of identity with the main group of bacterial transposases, the highest being 26 % identity between the ISTet2 and IS*26* transposases, raising questions of whether they should be in the same family as the main group found in bacterial species that includes IS*26* and IS*257/*IS4*31*.

### Properties of the IS*6*/IS*26* family members

To identify the most conserved features of the family members that are most similar to IS*26* and IS*257/*IS*431*, the 65 bacterial ISs in clades I to VI (Fig. 1) and the single outlier ISFal2 (Fig. 1) were compared. This includes 44 of Gram-negative (mainly Proteobacteria) origin, and 21 of Gram-positive (mainly Firmicute) origin that are members of the IS*6*/IS*26* family in the original and strictest sense. The median IS length is 820 bp and the median transposase length is 235 aa (Tables 3 and 4). The transposases of these shorter bacterial ISs share 28.9–94.6 % amino acid identity to one another, with members of clade III most diverged from members of clades I, II, IV, V and VI. Alignment of all transposases (Fig. S1) revealed that, in addition to the DDE residues that have been shown to be required for activity [40], they share many conserved amino acids. However, those from each clade share some specific features, e.g. clade VI has a well conserved 14 aa N-terminal extension.

**Fig. 3.**
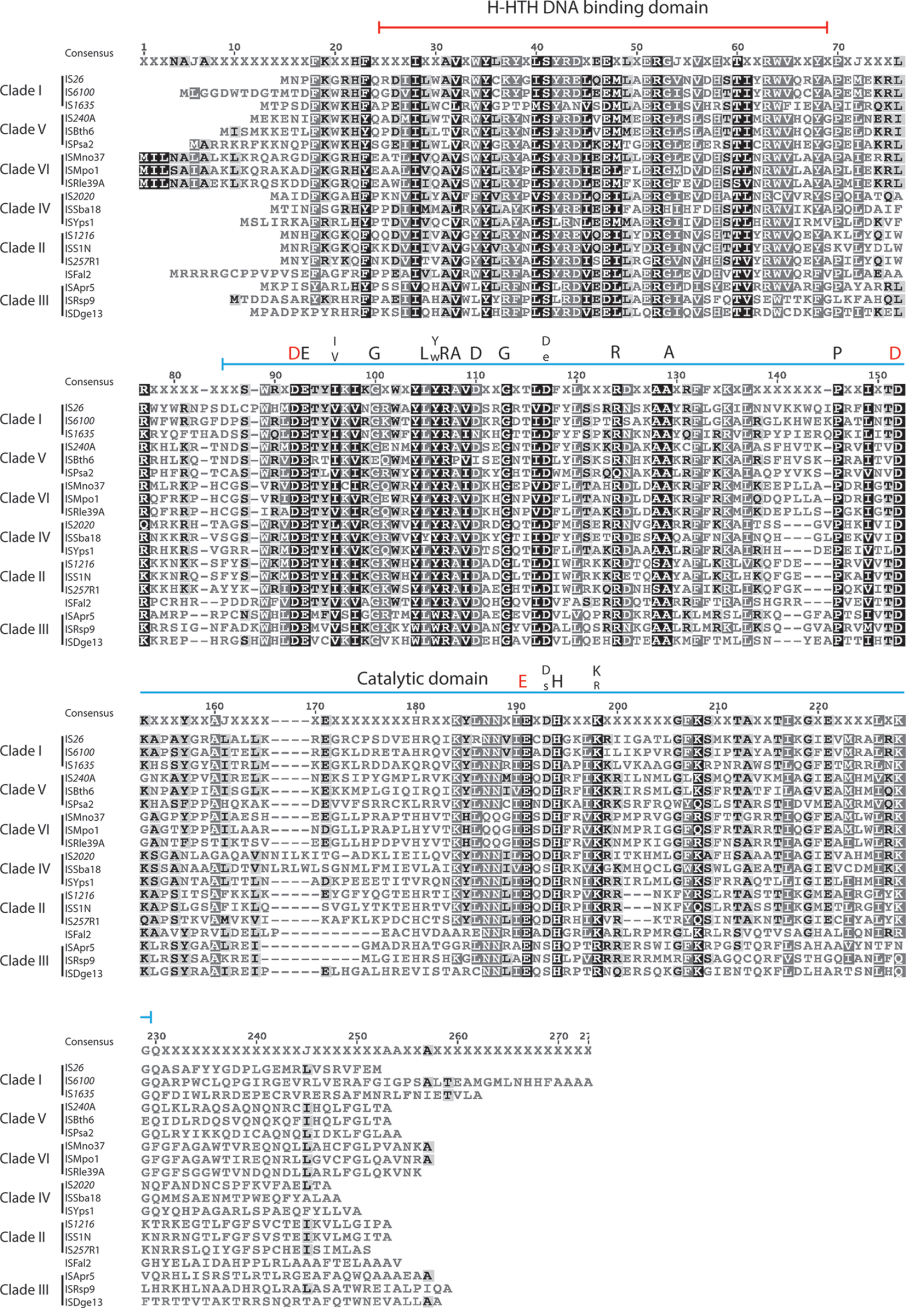
Alignment of the amino acid sequences of representative transposases of bacterial IS*6*/IS*26* family members. The transposases of three representatives from clades I to VI, and ISFal2 are shown. The extents of the H-HTH putative DNA binding domain and the DDE catalytic domain are marked above. Residues in the catalytic domain that are conserved in at least 62 of the 65 transposases in the complete alignment (Fig. S1) are marked with a capital letter. Residues for which only two variations exist are marked with smaller letters. The completely conserved DDE residues are marked by red letters. Amino acids are shaded as follows: black, 100 % similarity; dark grey, 80–99 % similarity; light grey, 60–79 % similarity; unshaded, less than 59 % similarity.

An alignment of 19 transposases selected to represent each clade and including the ISFal2 outlier is shown in Fig. 3. Near the N-terminus, significant conservation can be seen in a predicted helix-helix-turn-helix (H-HTH) domain. Two highly conserved planar residues, phenylalanine (F), and F or tyrosine (Y), separated by 4 aa found in a short segment at the extreme N-terminus are evident in five clades; some clade III members include only one of them. However, while clearly important, the function of this region is unknown at present. The spacing of the critical D and E residues in the catalytic domain, previously indicated to be DD(34)E [2] as in IS*26*, is 34 aa in clades I, V and VI, but 33 in clade II, longer in four members of clade IV and shorter in ISFal2 and clade III, which lies on the deepest branch in Fig. 1. However, several completely or highly conserved amino acids (no more than 3 differences in the full alignment in Fig. S1) can be seen in the catalytic domain and are marked above the alignment in Fig. 3. Notably, while conservation of a lysine (K) residue separated by 6 aa from the E of the DDE triad is seen in several DDE transposase families (including retroviral integrases [47]), in these IS*6*/IS*26* family transposases, a histidine (H) is also completely conserved and likely a signature for the family. The motif E-DH---K is seen in 55 Tnp belonging to five of the six clades, and E-SH---R (S=serine; R=arginine) is seen in 10 Tnp (9 in the most diverged clade, clade III). An E adjacent to the first D, often followed by threonine (T), and an additional D (or E in 2 cases) residue located between the D residues of the DDE motif are also seen in other DDE transposase families [2]. However, a completely conserved G (glycine) and the completely conserved P (proline) also located between the D residues are notable features unique to this group and, hence, potential signatures for this family.

**Fig. 4.**
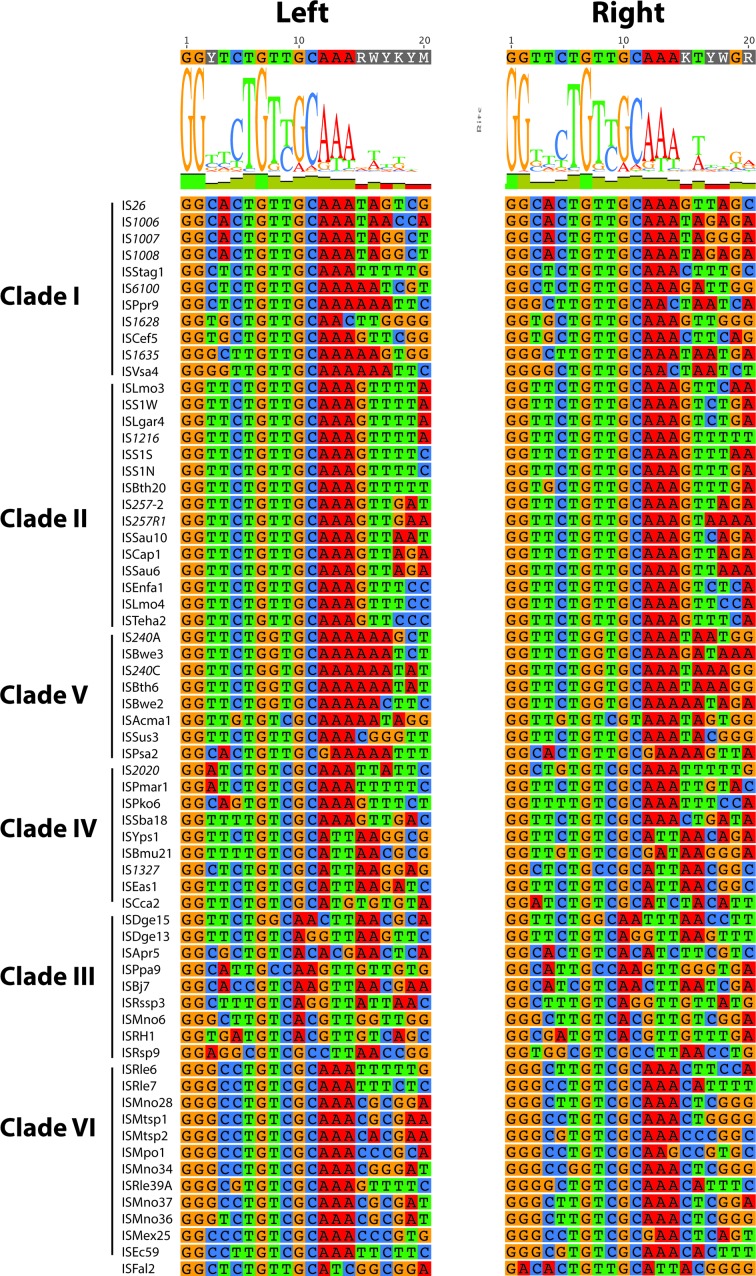
Alignment of the TIRs of the short bacterial IS*6*/IS*26* family members. The first 20 nucleotides and the complement of the last 20 nucleotides of each of the bacterial ISs from clades I to VI are aligned on the left and right, respectively, with each base coloured specifically. The consensus is shown above.

Though IS*26* has perfect 14 bp TIRs, the lengths of the TIRs, as defined in the ISFinder records, are quite variable and the 14 bp at the left and right ends of many ISs are not perfect inverted repeats. Alignment of the first and last 20 bp of each IS for the 65 shorter bacterial ISs captured the span of the majority of the reported TIRs, and revealed a strong 14 bp consensus (Fig. 4). Five ISs that in ISFinder did not both start and end with GG were re-examined using additional available sequences in GenBank, allowing the ends to be redefined by removing a single base at each end (Table S3). The final TIR consensus was **GG**TTCT**G** TTGCAAA at both ends (Fig. 4). The three bases in bold are conserved in all but the right TIR of ISFal2, which starts with GA, and the G at position 10 is also highly conserved but replaced by A in clade III. The most variation is seen at positions 3 and 4. The underlined sequence represents a −35 motif that is bi-directional and the outward facing configuration has frequently been seen coupled with a −10 motif in the adjacent DNA leading to increased expression of the adjacent gene(s) [36, 48–53].

This set of ISs includes the IS originally used to define the IS*6* family, and it represents the group we are defining as the true members of the IS*6*/IS*26* family.

### Properties of the longer bacterial transposases and ISs

**Fig. 5.**
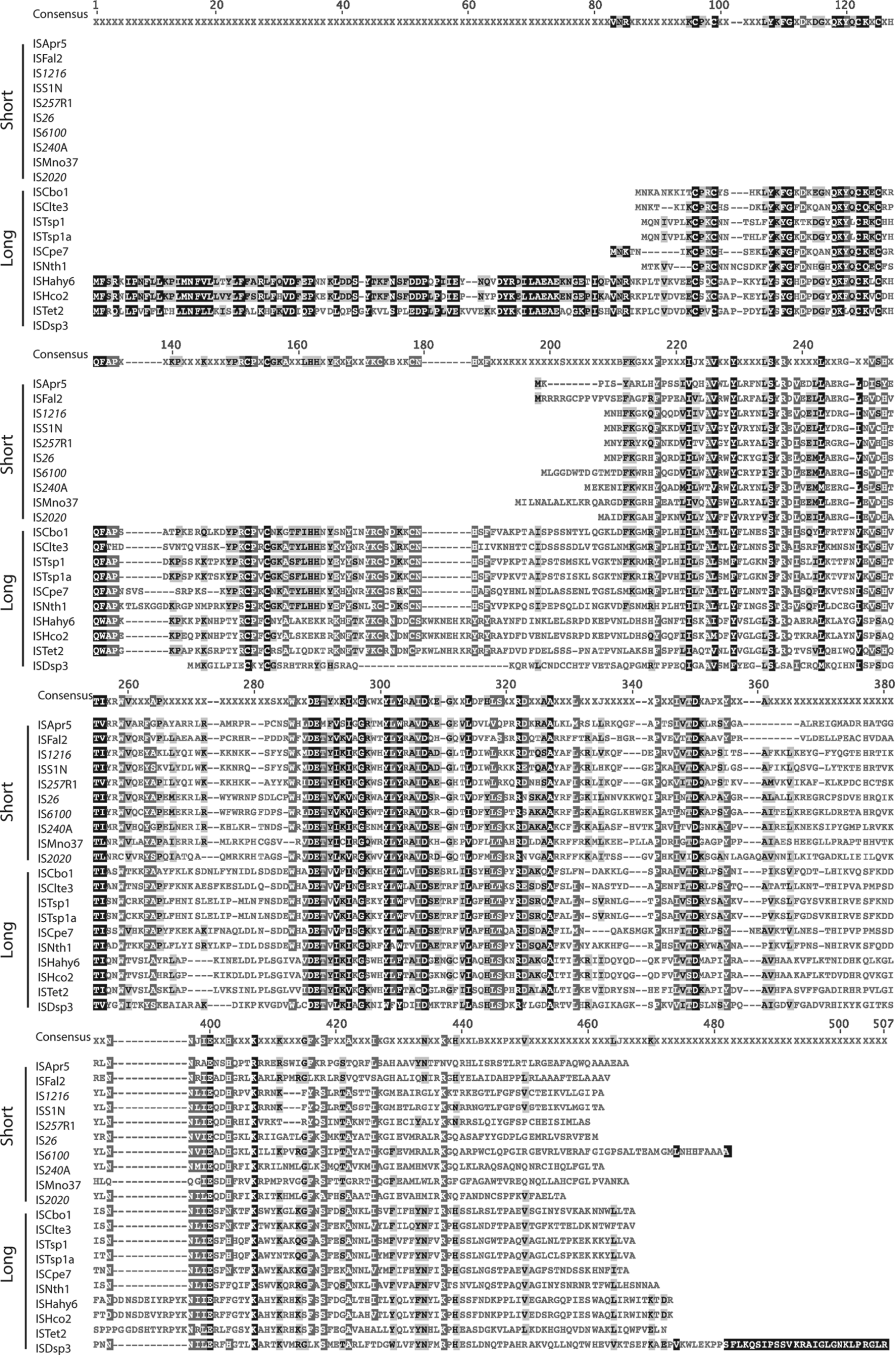
Alignment of the ten long ISs and ten representatives of the short bacterial ISs from clades I to VI. A ClustalW alignment (BLOSUM matrix, gap open cost 10, gap extend cost 0.1) was generated in Geneious (version 7.1.9) to align the transposases of the ten long bacterial ISs and ten representatives of the short bacterial ISs from clades I to VI. Amino acids are shaded as follows: black, 100 % similarity; dark grey, 80–99 % similarity; light grey, 60–79 % similarity; unshaded, less than 59 % similarity.

The long bacterial ISs are clearly separated from both the main bacterial group and from the archaeal group. They fell into two groups designated clades VII and VIII, with one outlier. The highest identity of a transposase from this group with a short bacterial transposase was 26 %, and they share 24 to 91 % identity with each other. However, an alignment of the encoded transposases with 10 representative short transposases from bacterial ISs (Fig. 5) revealed some similarities in the highly conserved amino acids residues of both the H-HTH domain and the catalytic domain, but also some differences. In particular, not all of the signature conserved amino acids in the catalytic domain of the small transposases outlined above were present. For example, the characteristic E-DH--K (or E-SH--R) is not present and the two conserved F(Y) at the extreme N-terminus of the short transposases are not reliably both present.

**Fig. 6.**
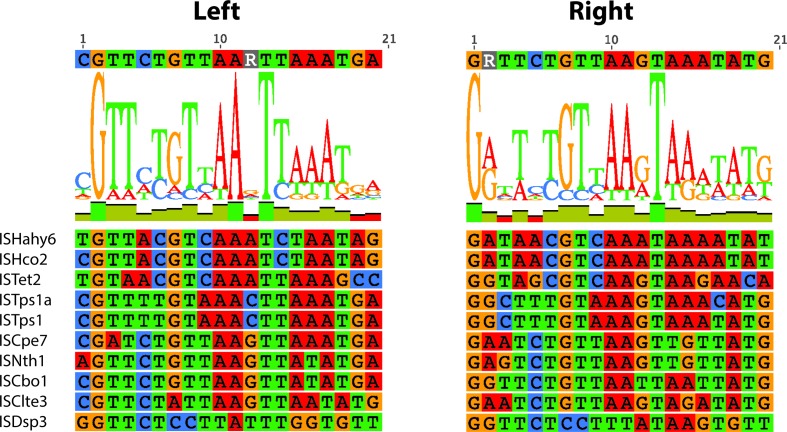
Alignment of the TIRs of the long bacterial IS*6*/IS*26* family members. The first 20 nucleotides and the complement of the last 20 nucleotides of each of the bacterial ISs from clades VII and VIII are aligned on the left and right, respectively, with each base coloured specifically. The consensus is shown above.

Comparison of the TIRs (Fig. 6) also revealed some similarities together with some marked differences. The consensus extended to 18 bp in contrast to the 14 bp consensus for the short bacterial ISs. The conserved G7 was present in most of them with G2 on the left and G1 on the right also highly conserved, but the G10 was not present. However, these bases are also found in the TIRs of other IS families [2, 8]. A striking difference is that the outermost bases of the TIRs are rarely complementary, and this is likely to have consequences for the movement mechanism(s), which remains to be established. Hence, the features do not support the placement of these ISs with the short bacterial ISs in the same IS*6*/IS*26* family. Indeed, their potential for assignment to another current DDE family should be considered or they should form a separate family with two groups defined by the length of the N-terminal extensions.

### Properties of the archaeal ISs

**Fig. 7.**
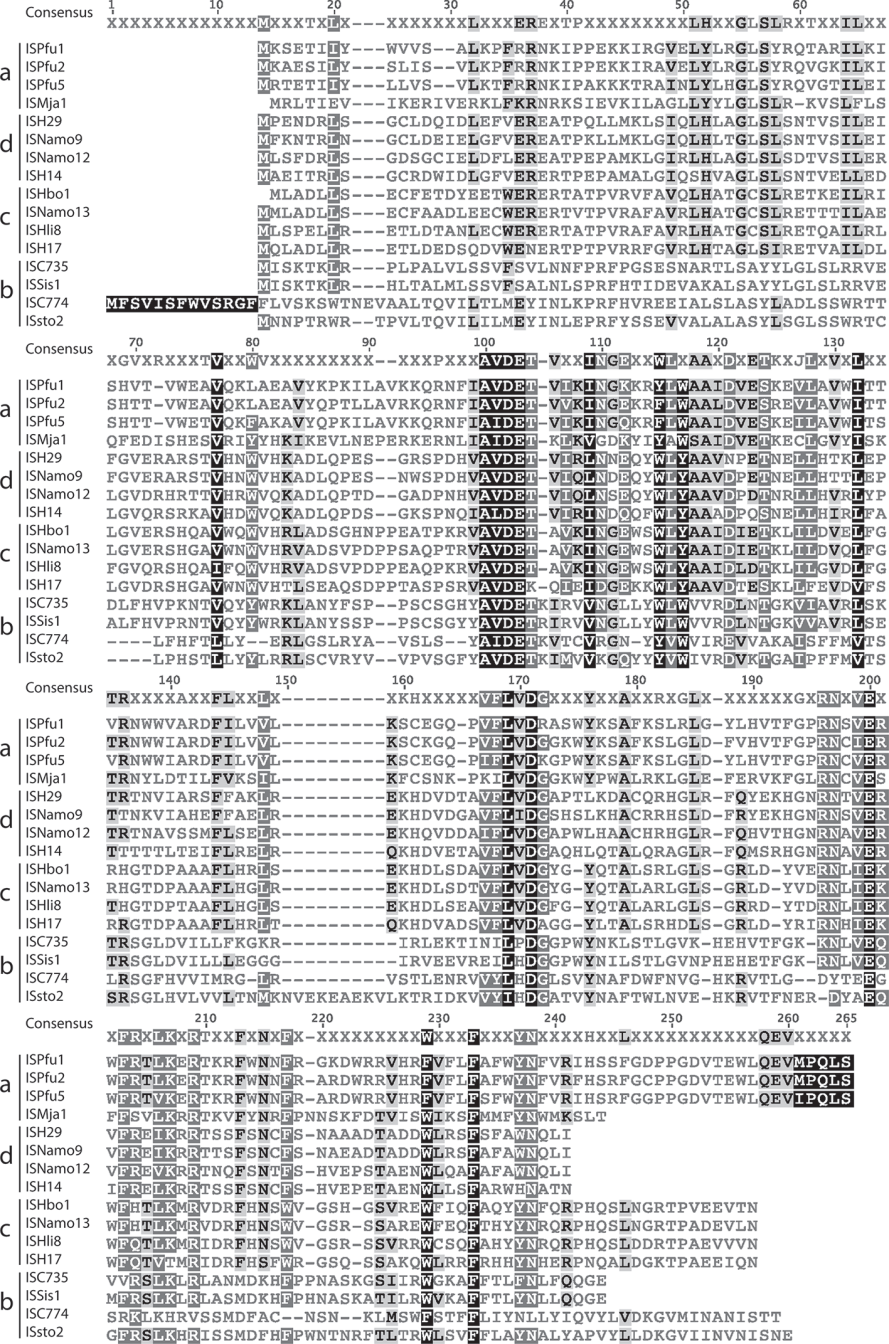
Alignment of representative transposases from archaeal IS*6/*IS*26* family members. The transposases of four representatives from archaeal clades a to d are shown. Amino acids are shaded as follows: black, 100 % similarity; dark grey, 80–99 % similarity; light grey, 60–79 % similarity; unshaded, less than 59 % similarity.

No ISs derived from archaea were assigned to this family in the original division into families [2]. Though the basis for their inclusion is not recorded, by 2007 14 archaeal ISs had been assigned to the family. They were described as belonging to three groups corresponding to the genus of the organism they were found in [54]. Here, the transposases of the 37 ISs isolated from archaea (Table 5) that are currently placed in the IS*6* family formed two distinct branches with two clades in each (Fig. 1). The alignment of four representative transposases from each clade is shown in Fig. 7 and the complete alignment is in Fig. S2. There is clear conservation within the catalytic domain, but the signature of conserved amino acids is not like that for the transposases in Figs 3 and S1. For example E-DH---K in the bacteria is replaced by E--F---K-R in the archaea, and there is clear conservation of large non-polar amino acids, W---F, at the C-terminal end of the catalytic domain not seen in this region for the short bacterial transposases. This reinforces the conclusion that the archaeal ISs should not be placed in the IS*6*/IS*26* family.

**Fig. 8.**
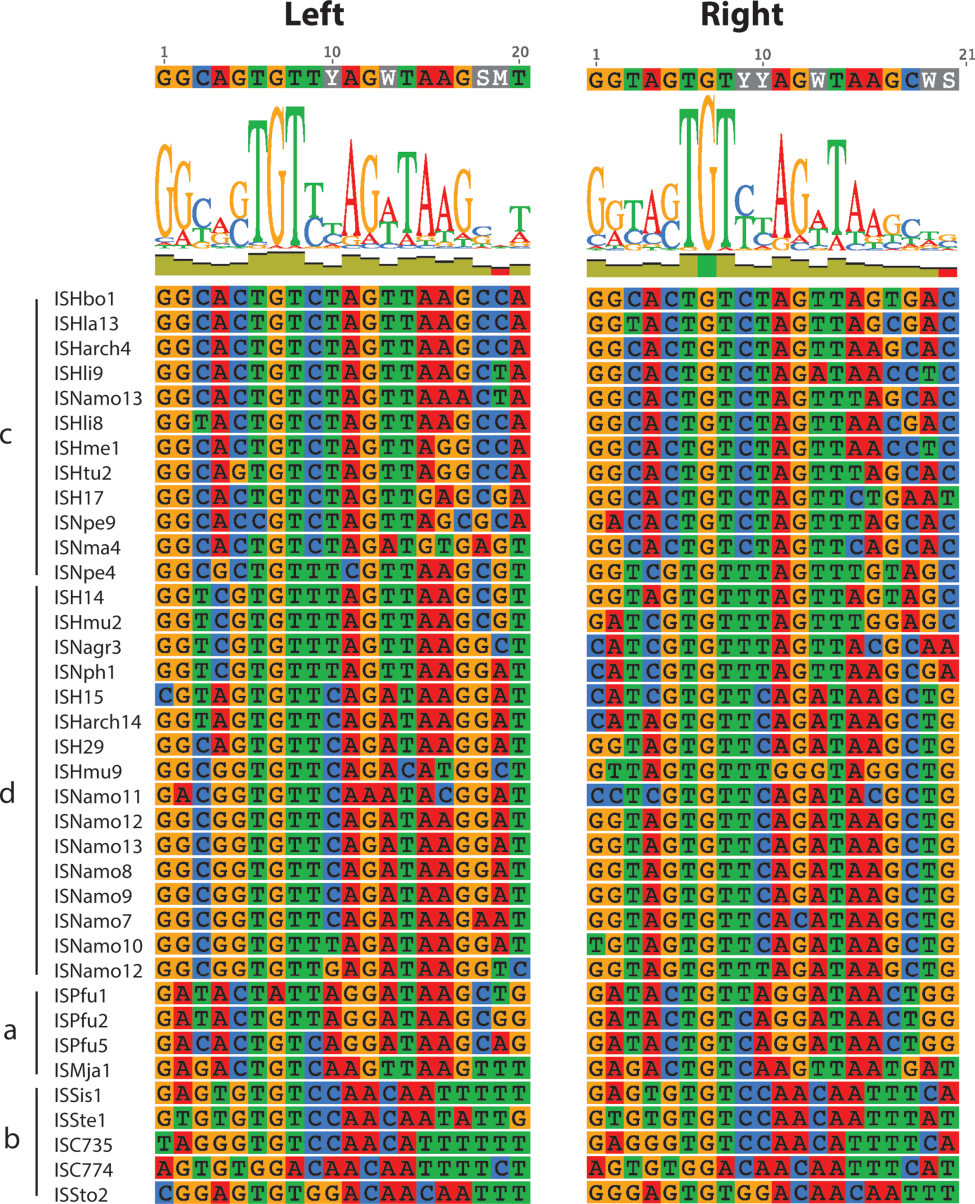
Alignment of the TIRs of the archaeal IS*6*/IS*26* family members. The first 20 nucleotides and the complement of the last 20 nucleotides of each of the archaeal ISs from clades a to d are aligned on the left and right, respectively, with each base coloured specifically. The consensus is shown above.

It was also noted previously that the outermost bases in the TIRs of these ISs were often not GG [54]. However, a current alignment (Fig. 8) shows that many in the expanded group analysed here start and/or end with GG and also include a G in position 7, as for the main bacterial group, but G10 is not present. This difference likely arises from the predominance of ISs from the two smaller clades (a and b) in the earlier set, whereas those in the other, now more populated, branch have ends that are similar to the bacterial consensus. However, the consensus for the archaeal TIR is longer, extending to at least 18 bp. As the sequences of many of the archaeal ISs have only ever been reported once, further examination of those that differ was not possible.

These findings are not consistent with the conclusion that these ISs should be in the IS*6*/IS*26* family. The possibility that they should form their own family needs to be considered.

## Discussion

The most surprising aspect of contemporary studies of IS*26* was the discovery of a novel conservative targeted mode of cointegrate formation that was the preferred reaction when two IS*26* were present in the same cell [40]. It is not yet known whether other ISs can utilize this additional mechanism and here we sought to identify the most likely IS candidates, namely those most closely related to IS*26*. These would clearly be those in clade I, followed by the ISs with transposases in clades II, IV, V and VI, which form the bulk of the IS6/IS*26* family as re-defined here. When movement of IS*26* was first examined, it was shown to utilize a replicative mechanism that generates cointegrates as the final product [18–22], and cointegrate formation, historically a hallmark of ISs in this family, has been demonstrated for at least one IS belonging to three of the five clades. These are IS*26* [20, 21, 25, 40, 55], IS*6100* [28, 32] and IS*1635* [35] in clade 1, IS*257* [30, 31], IS*214* (a synonym of IS*1216*) [34] and ISS1S [33] in clade II, and ISYsp1 [29] in clade IV. It seems possible that these ISs all share the mechanistic capabilities exhibited by IS*26* [37–40]. Though members of clade III, the most diverged of the six short bacterial clades, differ at a few of the positions in the transposase and the TIRs that are otherwise completely conserved, they have also been included in the re-defined IS*6*/IS*26* family. We have elected to refer to this family as the IS*6*/IS*26* family, because naming a family after an IS with no ISFinder entry seems inappropriate in the genomic era and, unfortunately, our efforts to obtain the original Tn*6*-containing plasmid JR72 in order to sequence it have been so far unsuccessful.

Knowledge of the completely conserved residues, identified via the alignment of the transposases and TIRs in this re-defined family, can now be used to examine their role in cointegrate formation and in determining whether a copy-in or conservative route is used. In particular, the role of the first 10 aa of the transposases, which includes two F (or Y) residues that are very well conserved, deserves attention. The conserved H near the conserved E (E-DH---K or E-SH--K) in the catalytic domain appears to be a signature for the family as it is not seen in other families (see figure 3 in the 1998 paper by Mahillon and Chandler [2]). For the IS*6*/IS*26* family, a 14 bp sequence at the outer ends is strongly conserved with complete conservation of G residues at bases 1, 2 and 7, except for one end of a single IS. The importance of G1 and G2 is established, as changing the GG at one or both ends of IS*26* to TT abolished copy-in and conservative cointegrate formation, respectively [39]. Whether the other conserved bases are essential for transposase binding and/or cointegrate formation can now be determined. However, we note that the feature GGnnnTG, which is present in most of these ISs, is shared by ISs in the IS*1* family (see table 1 in the 2015 paper by Siguier *et al*. [8]). Further analysis, particularly relating to the size of the TSD, is needed. Where recorded, it is 8 bp and in a few cases, such as for IS*26*, this has been shown experimentally. However, the duplication size is recorded in ISFinder for surprisingly few of the ISs in Table 3. Though this may be due to their activity causing deletions of adjacent DNA, thereby removing one copy of the duplication, it is also possible that the context of multiple copies has not been examined.

The ISFinder database is a repository for the DNA sequences and predicted encoded transposases of ISs and as such is an important resource, particularly in the genomic era. Classification into families is intended to identify groups of ISs that most resemble one another and, hence, are likely to share the same specific mechanism(s). Though defining families, or groups within families, is likely difficult when only sequence data are available, currently, it is also a non-transparent process. We have been unable to find any record of the criteria currently used when new ISs are submitted to ISFinder, and they should be clearly defined and available to all. The analysis of relationships within the single IS ‘family’ undertaken here highlights the possibility that they should be more stringent. The phylogeny distinguished two quite distinct types of transposase in addition to those of the original IS*6*/IS*26* group described above. The 10 ISs derived mainly from clostridial species are distinguished by the presence of one or more additional N-terminal domains in the transposase, and the ISs found in archaea form the second group. Each of these groups is at best distantly related to the other or to the re-defined IS*6*/IS*26* family and, as we did not find any experimental data for either the longer bacterial ISs or the archaeal ISs, this makes their inclusion with the original members of the family highly speculative. Consequently, we propose that they should be separated from the IS*6*/IS*26* family. Whether they should form new families or be included in other existing families must await information on an agreed set of criteria to be used to define families of ISs that use a DDE transposase.

Our analysis also revealed that some curation of the entries in ISFinder is needed. We recommend that the separately listed isoforms and synonyms should be collapsed into a single entry. This will be particularly important for IS*26* where this is causing unnecessary confusion in recent publications and GenBank submissions, as some copies are annotated as IS*26* and others as IS*15*D1 (this is IS15Δ1) and even as IS*6*. The known IS*26* variants have only a few base differences (see the 2019 paper by Pong *et al*. [44] for a recent analysis of the most abundant IS*26* variants). From an evolutionary perspective, the absence of significantly diverged isoforms of IS*26* is consistent with relatively recent dissemination, perhaps as antibiotic-resistance genes disseminated. In contrast, the IS*257/*IS*431* group includes several distinct isoforms, suggesting a long association with staphylococci. Most examples of IS*6*/IS*26* family ISs from Gram-positive bacteria are in clade II, consistent with long evolutionary history in these species, but clade V contained Gram-negative examples along with Gram-positive ones mainly from bacillus species. The latter may result from a more recent horizontal gene transfer event.

This study raises questions of which shared features are most important or indeed used in classification, and indicates the need for a more transparent definition of the criteria used to assign a novel IS to a family. Separation of the original group of ISs, now designated the IS*6*/IS*26* family, served to uncover shared features that will underpin future experimental studies.

## Data bibliography

1. Siguier P, Perochon J, Lestrade L, Mahillon J, Chandler M. ISFinder: the reference centre for bacterial insertion sequences. Nucleic Acids Res 34:D32–D36. https://www-is.biotoul.fr (2006).

## Supplementary Data

Supplementary File 1
