# Supplementary material for: An analysis of the IS6/IS26 family of insertion sequences: is it a single family?
Source: Microb Genom. 2019 Sep 5;5(9):e000291. doi: 10.1099/mgen.0.000291 (PMC6807381; doi:10.1099/mgen.0.000291)
Supplement: Supplementary File 1 [file mgen-5-291-s001.pdf]

SUPPLEMENTARY Material

Fig. S1

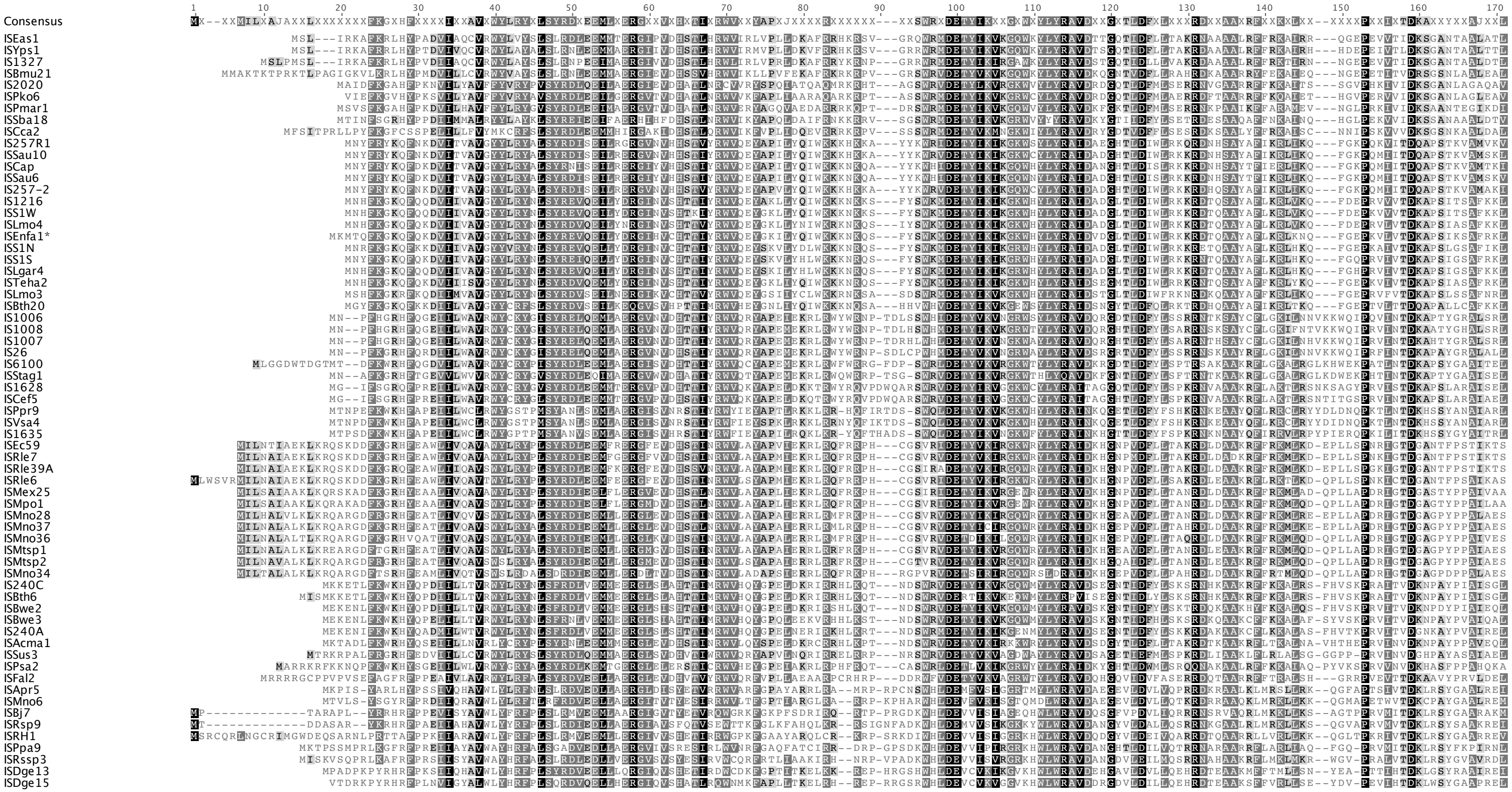

Fig. S1 cont.

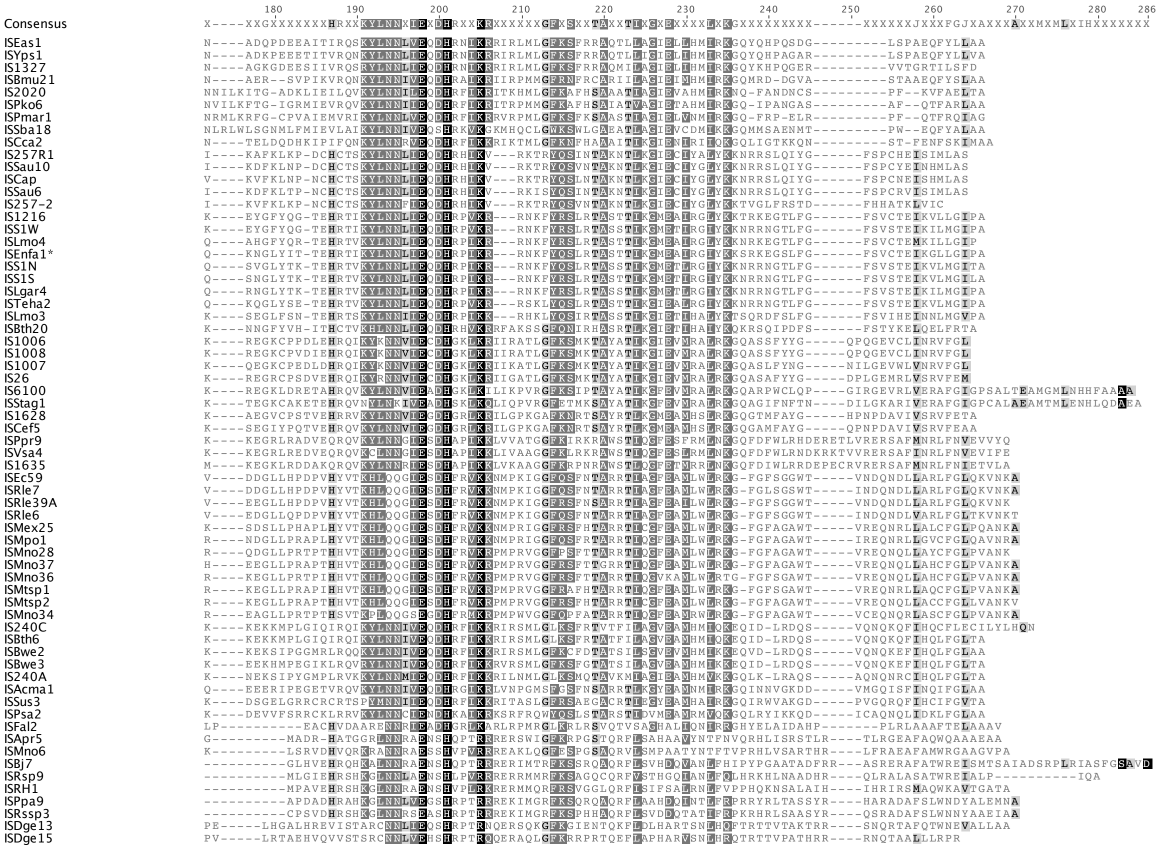

Fig. S1. Alignment of the transposases of the 65 short bacterial IS*6*/IS*26* family members. A ClustalW alignment (BLOSUM matrix, gap open cost 10, gap extend cost 0.1) was generated in Geneious (version 7.1.9) to align the complete set of short bacterial transposases. Amino acids are shaded as follows: black, 100% similarity, dark grey, 80-99% similarity; light grey, 60-79% similarity; unshaded, less than 59% similarity.

Fig. S2

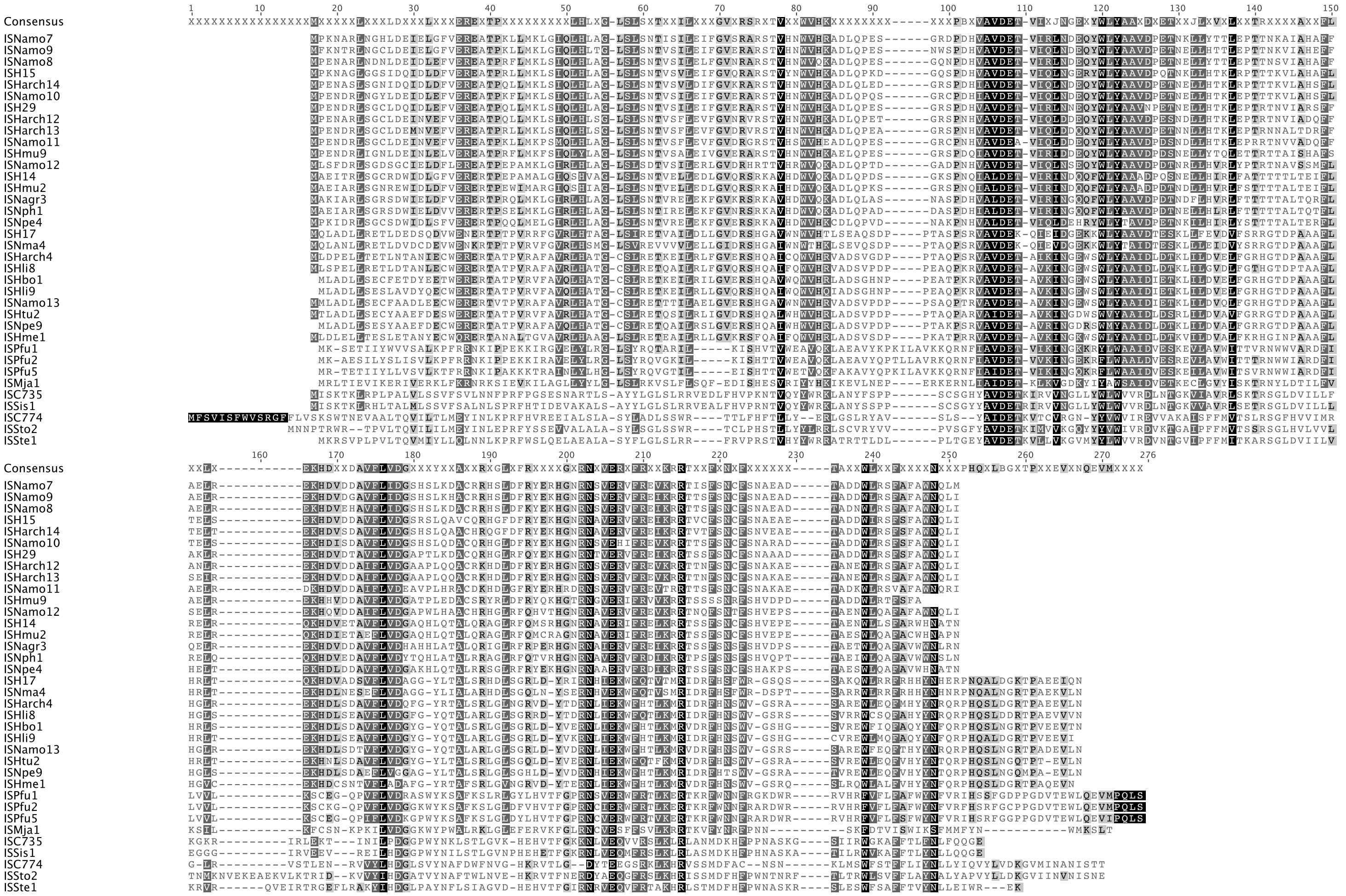

Fig. S2. Alignment of the transposases of the archaeal IS. A ClustalW alignment (BLOSUM matrix, gap open cost 10, gap extend cost 0.1) was generated in Geneious (version 7.1.9) to align the transposases of the archaeal IS classified as members of the IS*6* family in ISFinder. Amino acids are shaded as follows: black, 100% similarity, dark grey, 80-99% similarity; light grey, 60-79% similarity; unshaded, less than 59% similarity.

Table S1. IS isoforms

IS	Isoform(s)*
IS*26*†	IS*15*Δ1, IS*15*Δ2, IS*15*Δ4
IS*1008*	ISOur1
ISRle39A	ISRle39B, ISRle39D
IS257R1	IS*431*mec, IS*431*L, IS*431*R, IS*257*R2, IS*257*-1, IS*257*-3
IS*1216*‡	IS*1216*E, IS*1216*V
ISS1N	ISS1CH, ISS1D, ISS1E, ISS1M, IS*1297*
ISS1S	ISS1A, ISS1B, ISS1RS, ISS1T, ISS1X, ISS1Z, IS*946*M, IS*946*V
IS*240*A	IS*240*B

Isoforms defined using ISFinder criteria: >98% amino acid similarity and/or >95% nucleotide identity.

† IS*26* has been known by a number of different names; IS*15*Δ, IS*46*, IS*140*, IS*160*, IS*176,* IS*1936*.

‡ Previously also known as IS*214*.

Table S2. IS removed or modified

IS	Action	Justification
ISEnfa1	Modified	Truncated Tnp (C). Second sequence identified (KX579977), 10bp deletion in original entry.
ISSod8	Removed	Truncated Tnp (C), no other sequences available
ISAcr2	Removed	Truncated Tnp (C), no other sequences available
ISCgl3	Removed	Truncated Tnp (N), no other sequences available
ISCca8	Removed	Missing first D from DDE, M instead, no other sequences available
ISSpu17	Removed	Truncated Tnp (C), no other sequences available
ISMno27	Removed	Missing E from DDE, V instead, no other sequences available
ISMno35	Removed	Missing E from DDE, A instead, no other sequences available
ISCap	Added	Not in ISFinder. GenBank EF177828, bases 2-793.
ISSau10	Added	Not in ISFinder. GenBank FN390947, bases 1-793.
IS*240*F	Removed	Stop codon in the transposase.
ISCaa16	Removed	Missing D from DDE, no other sequences available
ISXne2	Removed	Missing E from DDE, no other sequences available.

Table S3. TIRs (and overall IS sequence) modified

IS	Action	Justification
ISEnfa1	Removal of 1x G at each end	Other sequenced examples (e.g. CP031028) only have 2x G, not 3x G.
ISCef5	Removal of 1x G at each end	Other sequenced examples (e.g. CP010451) only have 2x G, not 3x G.
ISCaa16	Removal of 1x G at each end	Only ever sequenced once, but extra G is accounted for by position in chromosome in sequence without this IS (CP001102).
ISAcma1	Removal of 1x G at each end	Other sequenced examples (e.g. CP000844) only have 2x G, not 3x G.
ISRH1	Removal of 1x C at each end	Only ever sequenced once, but extra C is accounted for by position in chromosome in sequence without this IS (NZ_PIQN00000000).
ISC774	Addition of 1x A at each end	Consensus was out of alignment, additional sequences available to support A at each end (e.g. CP033238, CP033236).
